# Draft Genome Sequences of Two *Pseudomonas* Strains That Are Able To Use Furan Derivatives as Their Sole Carbon Source

**DOI:** 10.1128/MRA.01131-19

**Published:** 2020-01-09

**Authors:** Carlos Farkas, Raúl A. Donoso, Carla Gárate-Castro, Pamela Villegas, Roberto E. Durán, Michael Seeger, Danilo Pérez-Pantoja

**Affiliations:** a Programa Institucional de Fomento a la Investigación, Desarrollo, e Innovación, Universidad Tecnológica Metropolitana, Santiago, Chile; b Center of Applied Ecology and Sustainability (CAPES), Santiago, Chile; c Laboratorio de Microbiología Molecular y Biotecnología Ambiental, Departamento de Química, Centro de Biotecnología Daniel Alkalay Lowitt, Universidad Técnica Federico Santa María, Valparaíso, Chile; University of Maryland School of Medicine

## Abstract

*Pseudomonas* sp. strains ALS1279 and ALS1131 were isolated from wastewater treatment facilities on the basis of their ability to use furfural, a key lignocellulose-derived inhibitor, as their only carbon source. Here, we present the draft genome sequences of both strains, which can shed light on catabolic pathways for furan compounds in pseudomonads.

## ANNOUNCEMENT

Lignocellulosic biomass such as corn stover and wheat straw can be used in biofuel production. However, their utilization as feedstocks requires pretreatments that generate by-products that inhibit microbial biocatalysts (e.g., furfural and 5-hydroxymethylfurfural [HMF]) (1, 2). Consequently, biodegradative routes for lignocellulose-derived inhibitors such as furan derivatives have attracted increasing interest due to their potential to be exploited in biodetoxification strategies to remove these compounds from hydrolysates (3, 4). Here, we report the draft genome sequences of *Pseudomonas* sp. ALS1279 and *Pseudomonas* sp. ALS1131, two strains that were isolated from wastewater treatment facilities on the basis of their ability to grow with furfural as their sole carbon and energy source (5). Moreover, we tested additional catabolic capabilities, which revealed that both strains were able to use furfuryl alcohol and furoic acid as growth substrates and that ALS1279 was also able to grow with HMF, HMF acid, or HMF alcohol as its sole carbon source.

**FIG 1:**
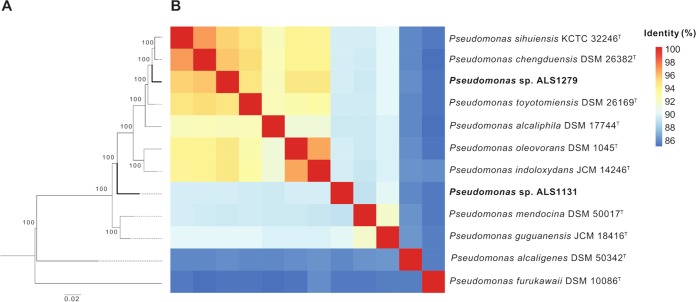
Phylogenetic analysis of 12 *Pseudomonas* sp. strains. (A) MLSA-based clustering of 12 *Pseudomonas* sp. strains, based on 31 housekeeping genes. Midrooted phylogeny showed distinctive clades for *Pseudomonas* sp. ALS1279 and *Pseudomonas* sp. ALS1131 isolates. (B) ANIm analysis of 12 *Pseudomonas* sp. strains. *Pseudomonas* sp. ALS1279 (ANIm, 95%) is part of cluster I, and *Pseudomonas* sp. ALS1131 (ANIm, 90%) is part of cluster III.

*Pseudomonas* sp. strains ALS1279 and ALS1131 were cultured in R2A broth (Neogen), and genomic DNA was obtained by using the GenElute bacterial genomic DNA kit (Sigma-Aldrich), for sequencing by MicrobesNG (Birmingham, UK) using Illumina MiSeq paired-end technology (2 × 250 bp). Libraries with a median insert size of 504 bp (ALS1279) or 487 bp (ALS1131) were generated using the Nextera XT library preparation kit (Illumina) following the manufacturer’s protocol. A total of 641,615 reads (ALS1279) and 1,224,184 reads (ALS1131) were obtained after sequencing and trimming using Trimmomatic v0.30 (6). Reads were assembled using SPAdes v3.9 (7), and assemblies were polished with two rounds of Pilon v1.23 (8). Default parameters were used for all software programs during bioinformatic analysis. The draft genome of *Pseudomonas* sp. ALS1279 consisted of 183 contigs (*N*_50_, 63,516 bp) and was 5,309,122 bp in size, with a G+C content of 62.5% and average coverage of 50×, while the genome of *Pseudomonas* sp. ALS1131 consisted of 43 contigs (*N*_50_, 256,635 bp) and was 5,564,837 bp in size, with a G+C content of 62.4% and average coverage of 89×. Gene annotation was performed using PGAP v4.7 (9) and indicated 4,737 coding proteins for ALS1279 and 5,078 coding proteins for ALS1131. Taxonomic classification for both strains was performed, as described previously (10), by combining multilocus sequence analysis (MLSA) and average nucleotide identity (ANI) analysis with JSpeciesWS v3.2 (11), using 10 publicly available genomes of type strains of *Pseudomonas* species. As depicted in Fig. 1, the strain most similar to *Pseudomonas* sp. ALS1279 was Pseudomonas chengduensis DSM 26382 (ANI using MUMmer average nucleotide identity [ANIm], 95.5%), suggesting that these strains are closely related (12). The strain closest to *Pseudomonas* sp. ALS1131 was Pseudomonas guguanensis JCM 18416 (ANIm, 90%); therefore, we were unable to identify this strain at the species level (12).

The genes involved in furfural biodegradation, encoding 2-furoyl-CoA synthetase (*hmfD*) (GenBank accession no. TRO33638 [ALS1131] and TRO30335 [ALS1279]), furoyl-CoA dehydrogenase (*hmfA*, *hmfB*, and *hmfC*) (GenBank accession no. TRO33635, TRO33636, and TRO33637 [ALS1131] and TRO30332, TRO30333, and TRO30334 [ALS1279]), and 2-oxoglutaryl-CoA hydrolase (*hmfE*) (GenBank accession no. TRO33639 [ALS1131] and TRO30336 [ALS1279]), were identified in both strains. In addition, specific genes for HMF degradation, encoding 2,5-furan-dicarboxylic acid decarboxylase (*hmfF* and *hmfG*) (GenBank accession no. TRO30339 and TRO33004), were identified only in ALS1279.

### Data availability.

The draft genome sequences of *Pseudomonas* sp. strains ALS1279 and ALS1131 have been deposited in GenBank under accession no. SCGA00000000 and SCGB00000000, respectively. Illumina sequencing reads are associated with BioProject no. PRJNA513496 and have been deposited in the SRA repository under accession no. SRR10040621 (ALS1279) and SRR10040622 (ALS1131). The versions described in this paper are the first versions of both genomes.
